# A novel adrenomedullin receptor signaling-based prognostic model predicts immunosuppressive microenvironment and informs therapeutic stratification in hepatocellular carcinoma: A retrospective bioinformatics study

**DOI:** 10.1097/MD.0000000000050066

**Published:** 2026-08-14

**Authors:** Dan Zhu, Siyi Zhong, Jiawei Hong, Chicheng Lu, Li Zhuang

**Affiliations:** a Department of Hepatobiliary and Pancreatic Surgery, Shulan (Hangzhou) Hospital, Shulan International Medical College, Zhejiang Shuren University, Hangzhou, P.R. China.

**Keywords:** adrenomedullin, hepatocellular carcinoma, immune microenvironment, machine learning, nomogram

## Abstract

The adrenomedullin receptor signaling pathway plays a crucial role in tumor progression, yet its comprehensive implication in hepatocellular carcinoma (HCC) remains underexplored. This study aimed to develop and validate a multi-gene prognostic signature based on adrenomedullin receptor signaling-related genes (ARGs) and to elucidate its associations with clinicopathological features, immune microenvironment, drug sensitivity, and somatic mutations in HCC. Transcriptomic and clinical data of HCC patients were obtained from The Cancer Genome Atlas and Gene Expression Omnibus databases. Pan-cancer analysis was performed to evaluate the expression and prognostic value of ARGs. A prognostic model was constructed using 116 machine learning algorithm combinations and evaluated by C-index and receiver operating characteristic analysis. The risk score (RS) derived from the model was further correlated with clinical characteristics, immune cell infiltration, drug sensitivity, somatic mutations, and pathway activities. A nomogram was established for clinical applicability. Single-cell RNA sequencing data were analyzed to delineate the cellular heterogeneity of ARG expression within the tumor microenvironment. *ADM* and *RAMP3* were significantly associated with HCC prognosis. The optimal model, built with Random Survival Forest, demonstrated robust predictive performance in both the Cancer Genome Atlas (HR = 9.51, *P* < .001) and GSE14520 (HR = 2.24, 95% CI: 1.46–3.43, *P* < .001) cohorts. High-risk patients exhibited advanced T stage, higher histological grade, and poorer overall survival. Computational immune profiling suggested decreased immune cell infiltration and altered immune checkpoint gene expression in the high-risk group. In silico drug sensitivity analysis predicted increased susceptibility to certain targeted agents (e.g., erlotinib) in high-risk patients, though these findings are hypothesis-generating and require experimental validation. Furthermore, RS was weakly correlated with tumor mutational burden (*R* = 0.16, *P* = .004), though TP53 mutation frequency did not differ significantly between risk groups after covariate adjustment. The nomogram integrating RS demonstrated favorable predictive accuracy for 1-, 3-, and 5-year survival. Single-cell analysis revealed predominant expression of ARGs in tumor endothelial cells. We developed and validated an ARG-based prognostic signature that effectively stratifies HCC patients into distinct risk subgroups. This model may serve as a hypothesis-generating framework for individualized prognosis prediction and for identifying potential therapeutic vulnerabilities in HCC that warrant prospective investigation.

## 1. Introduction

Hepatocellular carcinoma (HCC) remains a formidable global health challenge, ranking as the third leading cause of cancer-related mortality worldwide.^[1]^ The development and progression of HCC involve dysregulation of multiple signaling pathways that promote cell proliferation, angiogenesis, and metastasis, thereby complicating treatment efforts.^[2,3]^ Its pathogenesis is strongly associated with chronic liver inflammation and cirrhosis, frequently triggered by hepatitis B/C virus infection, alcohol abuse, and metabolic dysfunction-associated steatohepatitis.^[4,5]^ Despite advances in surgical techniques, locoregional therapies, and systemic treatments, such as tyrosine kinase inhibitors and immune checkpoint inhibitors, the prognosis for patients with advanced HCC remains poor, characterized by high rates of recurrence and metastasis.^[6,7]^ A major obstacle to improving outcomes is the high degree of heterogeneity in HCC, which is reflected in diverse molecular subtypes, clinical manifestations, and therapeutic responses.^[8,9]^ Thus, there is an urgent clinical need to identify robust molecular biomarkers that can improve patient stratification, enable accurate prognosis prediction, and reveal novel therapeutic vulnerabilities.

Adrenomedullin (AM) is a multifunctional peptide that plays important roles in physiological processes such as vascular homeostasis and blood pressure regulation.^[10]^ However, dysregulation of the AM signaling pathway: which involves receptors including the adrenomedullin-1 receptor and adrenomedullin-2 receptor, has been implicated in tumor promotion across multiple cancer types.^[10]^ AM activates the Gs-coupled calcitonin receptor-like receptor (*CALCRL*), triggering cAMP-PKA signaling cascades that modulate inflammatory responses.^[11]^ Within the tumor microenvironment, AM signaling facilitates cancer progression by promoting proliferation, angiogenesis, and metastasis.^[10,12]^ Notably, the AM-*CALCRL* axis has emerged as a therapeutic target of interest, as circulating AM levels may serve as diagnostic and prognostic biomarkers.^[13]^ Antagonizing AM receptors, particularly the adrenomedullin-2 receptor, represents a promising anticancer strategy since it may circumvent interference with AM’s physiological roles in blood pressure regulation.^[13]^ Although the specific function of AM signaling in HCC has not been extensively studied, its tumor-promoting mechanisms in other cancers suggest it may similarly contribute to HCC pathogenesis through regulating cell proliferation and metastatic behavior.^[13]^

In HCC, the dysregulation of several signaling pathways: such as TGF-β/SMAD, Wnt, PI3K/AKT, and FGFR4, has been shown to drive tumor progression.^[14–16]^ For example, TGF-β signaling activation can induce epithelial-mesenchymal transition and malignant phenotypes,^[17]^ while aberrant activation of the FGF19-FGFR4 pathway is recognized as a key oncogenic driver in approximately 30% of HCC cases.^[18]^ The interplay among these pathways underscores the need for multi-gene models that integrate various genetic markers, such as CCDC110 and TGFBR1, to improve prognostic accuracy. Previous studies have demonstrated that transcriptomic-based multi-gene signatures (e.g., MDH gene sets) can offer clinical guidance and help optimize treatment response.^[19]^ Therefore, developing a multi-gene prognostic model based on adrenomedullin receptor signaling-related genes (ARGs), in combination with other pathway genes, could enhance our understanding of the complex molecular mechanisms underlying HCC and provide a theoretical foundation for targeted therapies.

In this study, we curated a gene set related to adrenomedullin receptor signaling: designated as “GOBP_ADRENOMEDULLIN_RECEPTOR_SIGNALING_PATHWAY”: and constructed a multi-gene prognostic model for HCC. Using transcriptomic and clinical data from The Cancer Genome Atlas (TCGA, https://portal.gdc.cancer.gov/) and independent validation cohorts, we assessed the model’s ability to predict overall survival (OS), its correlation with clinicopathological features, and its relationship with the tumor immune microenvironment. Our work establishes a novel pathway-driven signature that improves risk stratification in HCC and may reveal actionable therapeutic targets within the AM receptor signaling pathway.

## 2. Materials and methods

### 2.1. Data acquisition and preprocessing

Transcriptomic data, clinicopathological information, survival outcomes, and somatic mutation data for the TCGA-LIHC cohort were obtained from The TCGA database. The transcriptomic dataset included 340 liver hepatocellular carcinoma (LIHC) samples, which served as the training-set for model construction. Additionally, transcriptomic and clinical data with follow-up information for the GSE14520 cohort were downloaded from the Gene Expression Omnibus (GEO, https://www.ncbi.nlm.nih.gov/geo/) database, comprising 221 HCC patients as an independent validation set. All expression data were in FPKM format and subsequently log2-transformed. Expression data from TCGA-LIHC (RNA-seq, FPKM, log2-transformed) and GSE14520 (microarray) were jointly subjected to ComBat batch correction (sva package, parametric empirical Bayes method) with dataset origin as the batch variable, followed by re-splitting into separate cohorts prior to model training. PCA plots before and after correction are provided in Supplementary Figure S1, Supplemental Digital Content 1 to demonstrate successful mitigation of inter-platform variability. Importantly, the risk score (RS) model was trained exclusively on the TCGA-LIHC portion of the batch-corrected data, and the GSE14520 portion was used solely as an independent validation set. Five ARGs: *ADM*, *ADM*2, *CALCRL*, *RAMP2*, and *RAMP3*: were retrieved from the MSigDB database (https://www.gsea-msigdb.org/gsea/msigdb/index.jsp) under the gene set GOBP_ADRENOMEDULLIN_RECEPTOR_SIGNALING_PATHWAY.

All data used in this study were obtained from publicly available databases (TCGA and GEO), which provide de-identified patient data in compliance with relevant data use agreements. As no new patient data were collected and no human subjects were directly involved in this study, formal ethics committee review was not required according to institutional policy. All analyses were conducted in compliance with the TCGA and GEO data use policies.

### 2.2. Pan-cancer analysis

Pan-cancer analyses were conducted using the TCGAplot package (v8.0.0). Gene expression patterns of ARGs across multiple cancer types were visualized via pan_boxplot, with statistical significance between tumor and normal tissues assessed using the Wilcoxon test. The association between ARG expression and OS was evaluated through pan_forest, which performed univariate Cox regression analysis.

### 2.3. Machine learning modeling

The Mime1 package (v0.0.0.9) was employed for model construction and evaluation. ARGs significantly associated with OS in the TCGA-LIHC training cohort were first identified via univariate Cox regression (unadjusted *P* < .05). Given the small number of candidate genes (n = 5), Bonferroni correction was applied (threshold *P* < .01). Only genes surviving this correction (*ADM* and *RAMP3*) were carried forward as input features for machine learning model construction. Clinical variables were not included as input features during model building but were evaluated in downstream univariate and multivariate Cox regression analyses. Subsequently, 116 machine learning algorithm combinations were applied to construct prognostic models under default parameters. Algorithm selection was performed using 10-fold cross-validation exclusively within the TCGA-LIHC training cohort, with the optimal model chosen based on the highest cross-validated concordance index (C-index). The GSE14520 cohort was strictly held out from all stages of algorithm selection and model building, serving solely as an independent external validation set. The selected model was used to compute an RS for each patient. Patients were stratified into high- and low-risk groups according to the median RS. Kaplan–Meier survival analysis with log-rank test was used to compare survival differences between groups. Time-dependent receiver operating characteristic (ROC) curves were generated to evaluate prediction accuracy for 1-, 3-, and 5-year OS. To assess model stability and potential overfitting, bootstrap resampling (1000 iterations) was performed within the training cohort to estimate the optimism-corrected C-index. Spearman correlation was used to assess the relationship between patient age and RS. Differences in RS across clinicopathological subgroups were compared using Wilcoxon or Kruskal–Wallis tests.

### 2.4. Immune microenvironment characterization

The IOBR package (v0.99.0) was utilized to evaluate the immune landscape. Infiltration levels of 10 immune cell types were estimated using the MCP-counter algorithm, which was selected because it is designed for bulk RNA-seq data and provides robust estimates of absolute immune cell populations without requiring tumor purity assumptions. ESTIMATE was additionally used to provide complementary stromal and immune scores, and immunophenoscore (IPS) was included because it has been specifically validated for immunotherapy response prediction. These 3 methods capture complementary aspects of the immune microenvironment. Differences between risk groups were compared via a Wilcoxon test. Spearman correlation between RS/ARGs and immune scores was computed and visualized via heatmaps. As a sensitivity analysis, immune infiltration estimates from MCP-counter were compared with those from CIBERSORT (absolute mode) and xCell. As these algorithms infer immune cell abundance from bulk gene expression profiles using reference signatures, they cannot capture spatial distribution, cellular activation states, or functional phenotypes of infiltrating immune cells. Results should therefore be interpreted as computational estimates rather than direct measurements of the tumor immune microenvironment.

### 2.5. Drug sensitivity analysis

Drug sensitivity was predicted using the pRRophetic package (v0.5). pRRophetic estimates half-maximal inhibitory concentration (IC50) values for individual samples by applying a ridge regression model trained on the Genomics of Drug Sensitivity in Cancer (GDSC) pharmacogenomic database, which comprises drug response profiles of cancer cell lines. Because cell line–derived pharmacogenomic models may not fully capture tumor heterogeneity, stromal interactions, or in vivo drug metabolism in patient populations, these computational predictions were used solely for exploratory purposes and should be interpreted as hypothesis-generating rather than clinically definitive. Spearman correlation analysis was performed between RS and drug sensitivity scores, and results were visualized. Wilcoxon test was used to compare drug sensitivity between high- and low-risk groups. As a complementary validation, key drug sensitivity predictions were cross-validated using the GDSC2 database via the oncoPredict package, and concordance between pRRophetic and oncoPredict predictions was assessed.

### 2.6. Somatic mutation analysis

Somatic mutation data in TCGA-LIHC were obtained from the GDC portal in MAF format, processed using MuTect2 as the variant caller. Synonymous mutations were excluded from tumor mutational burden (TMB) calculation, consistent with standard practice. TMB was defined as the total number of non-synonymous somatic mutations per megabase of the sequenced coding genome, calculated using the tmb() function in maftools (v2.24.0) with the genome size parameter set to 38 Mb. Variant filtering retained calls with a minimum sequencing depth of 10 × and minimum allele frequency of 5%. No additional harmonization was performed between risk groups as all samples were processed through the same TCGA pipeline. The coBarplot function was used to display mutation profiles between risk groups, and mafCompare was applied to identify differentially mutated genes. The comparison of mutation frequencies between risk groups used Fisher’s exact test with Bonferroni correction. TMB correlation with RS was assessed via Spearman analysis, and group differences in TMB were compared using the Wilcoxon test.

### 2.7. Differential expression and functional enrichment

Differentially expressed genes between risk groups were identified using the limma package (v3.64.1), with criteria of |log_2_(fold change)|>1 and adjusted *P*-value < .05. Functional enrichment analyses, including KEGG pathway and Gene Ontology analyses, were conducted using clusterProfiler (v4.16.0). Gene Set Enrichment Analysis was performed on Hallmark gene sets.

### 2.8. Construction and validation of a nomogram

Univariate and multivariate Cox regression analyses were used to identify prognostic factors in LIHC. In multivariate analysis, RS, T stage, histologic grade, and gender were included as separate covariates, with overall stage excluded to avoid multicollinearity with T stage. Clinical variables with more than 20% missing data were excluded from multivariate analysis; remaining missing values were handled by complete-case analysis. Independent predictors (*P* < .05) were incorporated into a nomogram using the rms package (v8.0–0). Calibration curves were generated via bootstrapping. Decision curve analysis (DCA) was performed using the rmda package (v1.6) to evaluate clinical utility. ROC analysis via the survival ROC package (v1.0.3.1) was used to assess predictive accuracy of the nomogram for 1-, 3-, and 5-year OS.

### 2.9. Single-cell expression analysis

Single-cell RNA sequencing (scRNA-seq) data from the GSE125449 dataset^[20]^ were reanalyzed to characterize ARG expression heterogeneity within the HCC tumor microenvironment. Raw count matrices generated by 10x Genomics Chromium were processed using the Seurat package (v5.0). Quality control filtering retained cells with nFeature_RNA ≥ 500, nCount_RNA > 700, and percent.mt < 20%; the permissive mitochondrial threshold reflects the known high mitochondrial content of hepatocytes and HCC cells. Doublet removal was performed per sample using DoubletFinder^[21]^ with an expected doublet rate of 7.5%, and only singlets were retained. Samples were merged and subsetted to HCC-derived cells based on author-curated annotations. Following normalization (LogNormalize, scale factor = 10,000) and identification of 2000 highly variable genes, PCA was performed with 20 principal components retained. Inter-sample batch effects were corrected using Harmony^[22]^ with sample identity as the batch covariate. UMAP was adopted as the primary visualization method for its superior preservation of global data structure, with t-SNE provided as a supplementary comparison. Graph-based clustering (resolution = 0.3) was applied to the Harmony-corrected embeddings. Six cell populations were annotated using canonical marker genes (T cells: CD3D/CD3E; B cells: CD79A; TECs: PECAM1/CDH5; CAFs: COL1A2/DCN; tumor-associated macrophages: CD14/CD68; hepatic progenitor cells (HPCs): EPCAM/KRT19), and validated by automated prediction using SingleR^[23]^ with the Human Primary Cell Atlas reference. Concordance between manual and automated annotations was assessed as supplementary material. ARG pathway activity scores were calculated using AddModuleScore, and differential expression and module score comparisons across cell types were evaluated by Wilcoxon rank-sum test.

### 2.10. Statistical analysis

All statistical analyses were performed using R software (version 4.4.0; R Foundation for Statistical Computing, Vienna, Austria). Continuous variables were compared between groups using the Wilcoxon rank-sum test (two groups) or Kruskal–Wallis test (three or more groups). Categorical variables were compared using Fisher’s exact test. Spearman rank correlation was used to assess monotonic associations between continuous variables. Potential sources of bias in this study include selection bias inherent to publicly available cancer genomics datasets, which may not be representative of the general HCC population. Misclassification bias may arise from computational inference of immune cell infiltration and drug sensitivity. Confounding by etiology, treatment history and liver function was not fully addressable due to data limitations. All multiple comparisons were corrected using the Benjamini–Hochberg procedure, and a false discovery rate threshold of *q* < .05 was applied unless otherwise stated.

## 3. Results

### 3.1. Pan-cancer expression and prognostic relevance of ARGs

**Figure 1.**
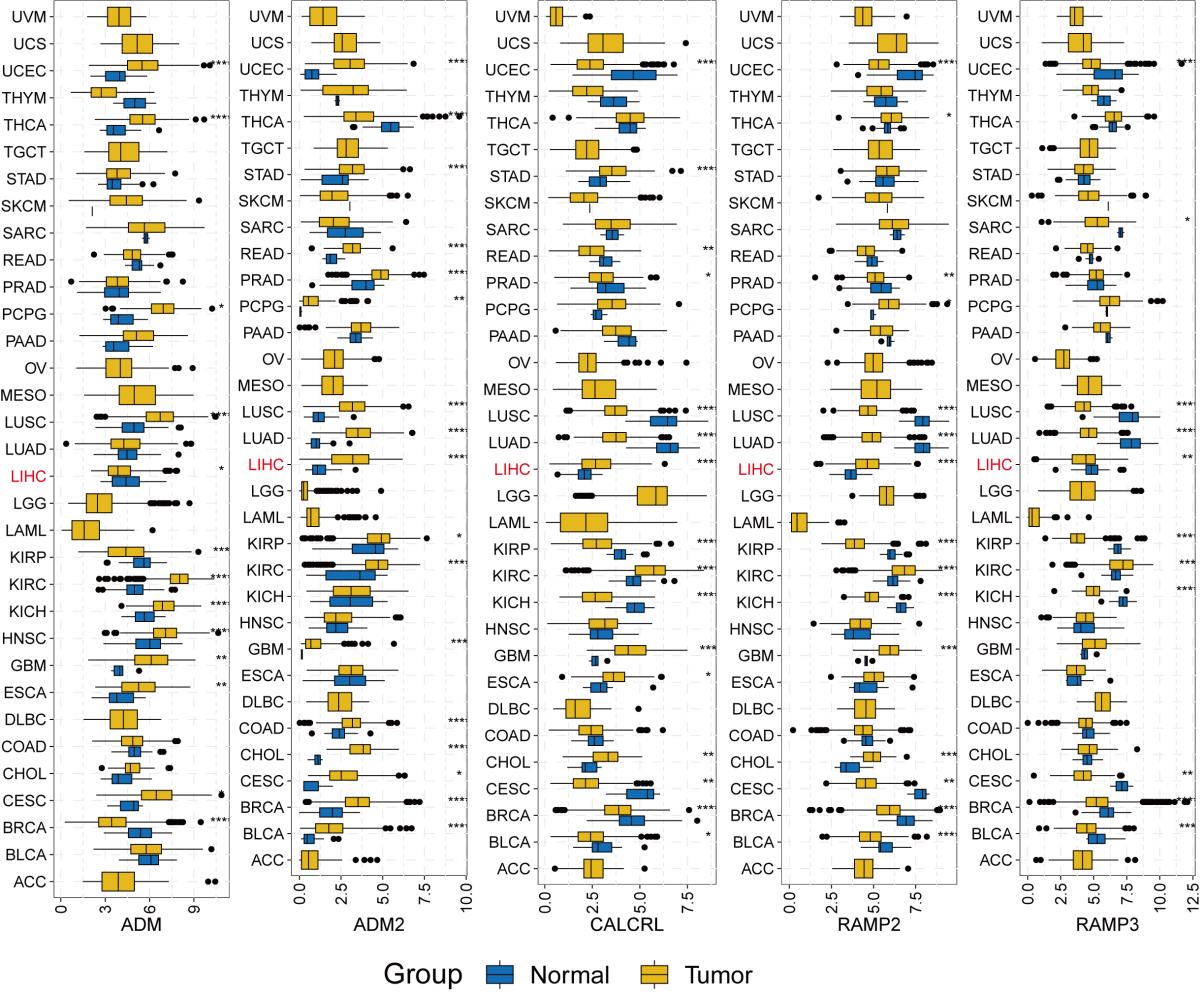
Pan-cancer expression of ARGs. Boxplots compare the expression of *ADM*, *ADM*2, *CALCRL*, *RAMP2* and *RAMP3* between tumor and normal tissues across TCGA cohorts. Statistical significance was determined by the Wilcoxon rank-sum test. *P < .05; **P < .01; ***P < .001.

We first performed a pan-cancer survey of the 5 adrenomedullin receptor signaling pathway genes (ARGs: *ADM*, *ADM*2, *CALCRL*, *RAMP2*, *RAMP3*) across TCGA cohorts. *ADM* expression was significantly elevated in UCEC, THCA, PCPG, LUSC, KICH, HNSC, GBM and ESCA but decreased in LIHC, KIRP and BRCA. *ADM*2 was downregulated only in THCA yet upregulated in 16 cancer types including UCEC, STAD and READ. *CALCRL* showed reduced expression in UCEC, READ and LUSC and increased expression in STAD, LIHC and KIRC. *RAMP2* was downregulated in UCEC, PRAD and LUSC but upregulated in THCA, KIRC and LIHC, whereas *RAMP3* was downregulated in UCEC, SARC and LUSC and upregulated in KIRC (Fig. 1).

In univariate Cox regression across TCGA cancers (Table 1), high *ADM* expression was associated with poorer OS in ACC, LGG, MESO, PAAD, SARC, THCA and UVM, but with better OS in KIRC and LUAD. Elevated *ADM*2 correlated with increased risk in CESC, HNSC, KICH, LGG, LIHC, LUAD, MESO and PAAD, but with decreased risk in GBM. *CALCRL* overexpression predicted worse OS in BLCA and LAML, yet better OS in GBM, KIRC and LGG. *RAMP2* upregulation was linked to higher risk in ACC, BLCA, MESO and STAD and to lower risk in HNSC, KIRC, PAAD and UVM. High *RAMP3* indicated favorable prognosis in HNSC, KIRC, LIHC and PAAD.

**Table 1:** Pan-cancer expression and prognostic analysis of ARSGs.

Cancer	*ADM*		*ADM*2		*CALCRL*		*RAMP2*		*RAMP3*	
	HR (95%CI)	*P* Value	HR (95%CI)	*P* Value	HR (95%CI)	*P* Value	HR (95%CI)	*P* Value	HR (95%CI)	*P* Value
ACC	1.398 (1.025–1.905)	.034	1.188 (0.944–1.497)	.143	1.298 (0.826–2.004)	.259	1.592 (1.052–2.409)	.028	1.068 (0.795–1.434)	.663
BLCA	1.033 (0.884–1.206)	.686	1.008 (0.885–1.147)	.906	1.312 (1.071–1.606)	.009	1.279 (1.061–1.542)	.01	1.093 (0.927–1.289)	.291
BRCA	1.033 (0.852–1.251)	.743	1.013 (0.872–1.178)	.865	1.147 (0.946–1.391)	.164	0.839 (0.698–1.01)	.063	0.893 (0.777–1.026)	.111
CESC	0.901 (0.709–1.146)	.397	1.309 (1.084–1.579)	.005	1.131 (0.868–1.474)	.361	0.786 (0.576–1.072)	.128	0.784 (0.599–1.028)	.078
CHOL	0.654 (0.342–1.252)	.2	1.156 (0.641–2.083)	.63	1.41 (0.853–2.332)	.181	1.174 (0.612–2.248)	.63	1.038 (0.683–1.578)	.86
COAD	1.272 (0.909–1.78)	.161	1.128 (0.878–1.449)	.347	0.975 (0.751–1.266)	.849	1.129 (0.844–1.509)	.415	1.042 (0.768–1.415)	.79
DLBC	1.462 (0.415–5.152)	.555	0.978 (0.491–1.949)	.95	1.125 (0.439–2.881)	.806	0.925 (0.261–3.277)	.904	1.402 (0.395–4.973)	.601
ESCA	0.99 (0.751–1.304)	.942	1.176 (0.987–1.402)	.07	1.165 (0.893–1.52)	.26	1.066 (0.81–1.403)	.647	1.009 (0.773–1.316)	.948
GBM	0.912 (0.726–1.147)	.432	1.127 (1.007–1.261)	.038	0.841 (0.717–0.987)	.034	0.993 (0.775–1.272)	.957	1.081 (0.947–1.233)	.248
HNSC	0.98 (0.853–1.125)	.77	1.19 (1.039–1.363)	.012	0.966 (0.825–1.132)	.671	0.788 (0.662–0.937)	.007	0.833 (0.72–0.964)	.014
KICH	1.104 (0.666–1.83)	.702	2.294 (1.206–4.363)	.011	0.813 (0.403–1.638)	.562	0.712 (0.278–1.825)	.479	0.821 (0.39–1.731)	.605
KIRC	0.837 (0.739–0.948)	.005	0.872 (0.753–1.01)	.067	0.713 (0.636–0.8)	0	0.702 (0.61–0.808)	0	0.685 (0.613–0.764)	0
KIRP	0.833 (0.67–1.036)	.1	1.238 (0.999–1.534)	.051	1.204 (0.899–1.611)	.213	1.126 (0.831–1.526)	.444	0.982 (0.719–1.342)	.91
LAML	0.837 (0.651–1.075)	.163	1.139 (0.944–1.376)	.175	1.338 (1.159–1.544)	0	0.935 (0.685–1.277)	.672	1.12 (0.795–1.578)	.516
LGG	2.28 (1.747–2.975)	0	1.208 (1.047–1.394)	.01	0.735 (0.642–0.841)	0	1.126 (0.805–1.577)	.488	1.134 (0.797–1.614)	.093
LIHC	1.065 (0.918–1.236)	.403	1.266 (1.077–1.488)	.004	0.965 (0.795–1.172)	.718	0.947 (0.776–1.155)	.589	0.798 (0.669–0.951)	.012
LUAD	0.812 (0.684–0.962)	.016	1.266 (1.117–1.436)	0	1.006 (0.849–1.191)	.947	0.95 (0.78–1.156)	.606	0.916 (0.771–1.089)	.319
LUSC	0.94 (0.814–1.087)	.405	0.957 (0.857–1.068)	.429	1.013 (0.856–1.199)	.877	1.02 (0.836–1.245)	.845	1.062 (0.882–1.278)	.525
MESO	1.658 (1.211–2.271)	.002	1.385 (1.116–1.655)	0	1.13 (0.921–1.385)	.241	1.35 (1.056–1.725)	.017	1.236 (0.981–1.558)	.073
OV	1.035 (0.896–1.197)	.638	1 (0.9–1.112)	.993	0.989 (0.835–1.171)	.896	1.018 (0.882–1.174)	.808	1.024 (0.849–1.234)	.804
PAAD	1.307 (1.039–1.645)	.022	1.24 (1.058–1.453)	.008	0.987 (0.792–1.23)	.907	0.701 (0.521–0.944)	.019	0.743 (0.566–0.977)	.033
PCPG	0.621 (0.167–2.316)	.478	0.606 (0.315–1.168)	.135	0.675 (0.311–1.464)	.32	0.584 (0.218–1.561)	.283	0.554 (0.259–1.182)	.127
PRAD	1.802 (0.661–4.912)	.25	0.922 (0.486–1.75)	.805	1.061 (0.504–2.235)	.876	0.468 (0.174–1.262)	.134	0.606 (0.248–1.479)	.271
READ	0.91 (0.407–2.034)	.818	0.638 (0.282–1.442)	.28	0.759 (0.373–1.546)	.448	1.454 (0.6–3.525)	.407	0.988 (0.449–2.175)	.976
SARC	1.236 (1.043–1.464)	.014	1.175 (1.025–1.348)	.02	0.97 (0.814–1.156)	.733	0.959 (0.794–1.157)	.66	0.735 (0.623–0.868)	0
SKCM	0.962 (0.851–1.088)	.538	1.031 (0.931–1.141)	.558	0.991 (0.861–1.139)	.895	1.042 (0.908–1.195)	.562	0.95 (0.832–1.085)	.451
STAD	1.117 (0.904–1.381)	.306	1.19 (1.008–1.405)	.04	1.217 (0.968–1.529)	.093	1.334 (1.042–1.708)	.022	1.299 (1.001–1.687)	.05
TGCT	1.231 (0.341–4.437)	.751	0.873 (0.341–2.234)	.777	1.212 (0.395–3.719)	.737	2.658 (0.677–10.426)	.161	0.813 (0.3–2.206)	.685
THCA	1.522 (1.072–2.162)	.019	0.842 (0.539–1.316)	.451	1.301 (0.778–2.173)	.316	1.013 (0.561–1.828)	.966	0.812 (0.461–1.433)	.473
THYM	1.107 (0.648–1.89)	.711	1.876 (1.048–3.358)	.034	0.568 (0.237–1.36)	.205	1.241 (0.514–2.996)	.631	0.892 (0.331–2.4)	.821
UCEC	1.232 (0.968–1.566)	.09	0.957 (0.783–1.169)	.665	1.247 (0.943–1.651)	.122	1.099 (0.791–1.528)	.574	0.848 (0.657–1.095)	.206
UCS	0.693 (0.442–1.086)	.109	1.237 (0.955–1.602)	.106	1.094 (0.801–1.492)	0.573	0.888 (0.634–1.244)	.49	0.963 (0.725–1.279)	.794
UVM	2.597 (1.518–4.445)	0	1.107 (0.602–2.038)	.743	2.799 (1.171–6.69)	.021	0.426 (0.202–0.9)	.025	1.089 (0.505–2.349)	.828

### 3.2. Construction and validation of an ARG-based prognostic model in LIHC

**Figure 2.**
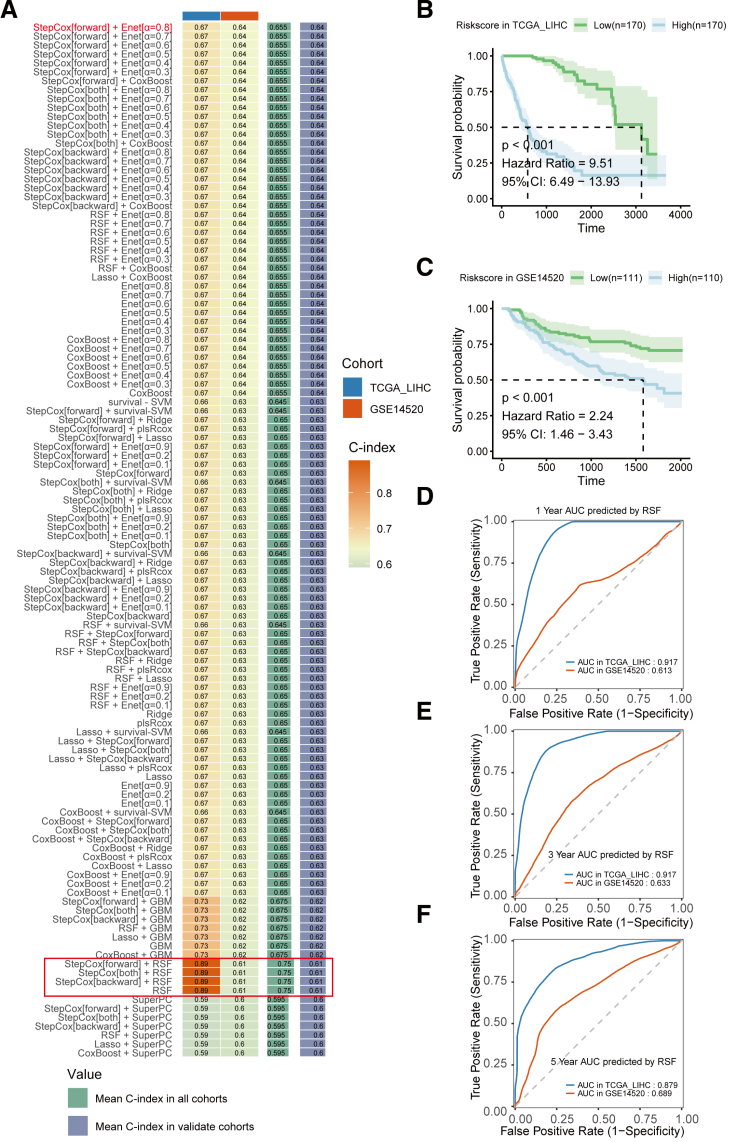
Construction and evaluation of the ARG-based prognostic model in LIHC. (A) Heatmap showing C-indices of 116 machine learning algorithm combinations evaluated via 10-fold cross-validation within the TCGA-LIHC training cohort. The selected model (RSF, highlighted) was subsequently validated in the independent GSE14520 cohort. (B–C) Kaplan–Meier survival curves for high- and low-risk groups in (B) TCGA-LIHC (HR = 9.51, 95% CI: 6.49–13.93, *P* < .001) and (C) GSE14520 (HR = 2.24, 95% CI: 1.46–3.43, *P* < .001). (D–F) Time-dependent ROC curves for (D) 1-year (AUC = 0.917/0.613), (E) 3-year (AUC = 0.917/0.633) and (F) 5-year (AUC = 0.879/0.689) OS in TCGA-LIHC/GSE14520. ARGs = adrenomedullin receptor signaling-related genes, LIHC = liver hepatocellular carcinoma, ROC = receiver operating characteristic, RSF = Random Survival Forest, TCGA = The Cancer Genome Atlas-LIHC = liver hepatocellular carcinoma.

Given their dysregulated expression and prognostic significance in LIHC, *ADM* and *RAMP3* were selected to build a multi-gene prognostic model. We evaluated 116 machine learning algorithm combinations using C-index as the performance metric. To avoid data leakage, the optimal algorithm was selected based exclusively on C-index computed within the TCGA-LIHC training cohort via 10-fold cross-validation, with the GSE14520 cohort strictly held out for independent external validation. Four Random Survival Forest (RSF)-based algorithm combinations (RSF, StepCox [forward] + RSF, StepCox [both] + RSF, StepCox [backward] + RSF) tied for the highest training-set C-index of 0.89, and RSF was selected as the representative final model (Fig. 2A). Patients were dichotomized into high-risk (High) and low-risk (Low) groups by the median RS. Kaplan–Meier analysis demonstrated that the High group had significantly shorter OS than the Low group in both TCGA-LIHC (HR = 9.51, 95% CI: 6.49–13.93, *P* < .001; Figure 2B) and GSE14520 (HR = 2.24, 95% CI: 1.46–3.43, *P* < .001; Figure 2C). Time-dependent ROC analysis yielded area under the curve (AUC) values of 0.917 and 0.613 for 1-year OS, 0.917 and 0.633 for 3-year OS, and 0.879 and 0.689 for 5-year OS in the TCGA-LIHC and GSE14520 cohorts, respectively (Figs. 2D–F). To quantify model optimism, bootstrap resampling (1000 iterations) was performed within the TCGA-LIHC training cohort, yielding an optimism-corrected C-index of 0.79 (95% CI: 0.74–0.83) compared with the apparent C-index of 0.89 (optimism = 0.10). External validation in GSE14520 yielded a bootstrap C-index of 0.58 (95% CI: 0.53–0.62; apparent C-index: 0.61).

### 3.3. Association between RS and clinicopathological features

**Figure 3.**
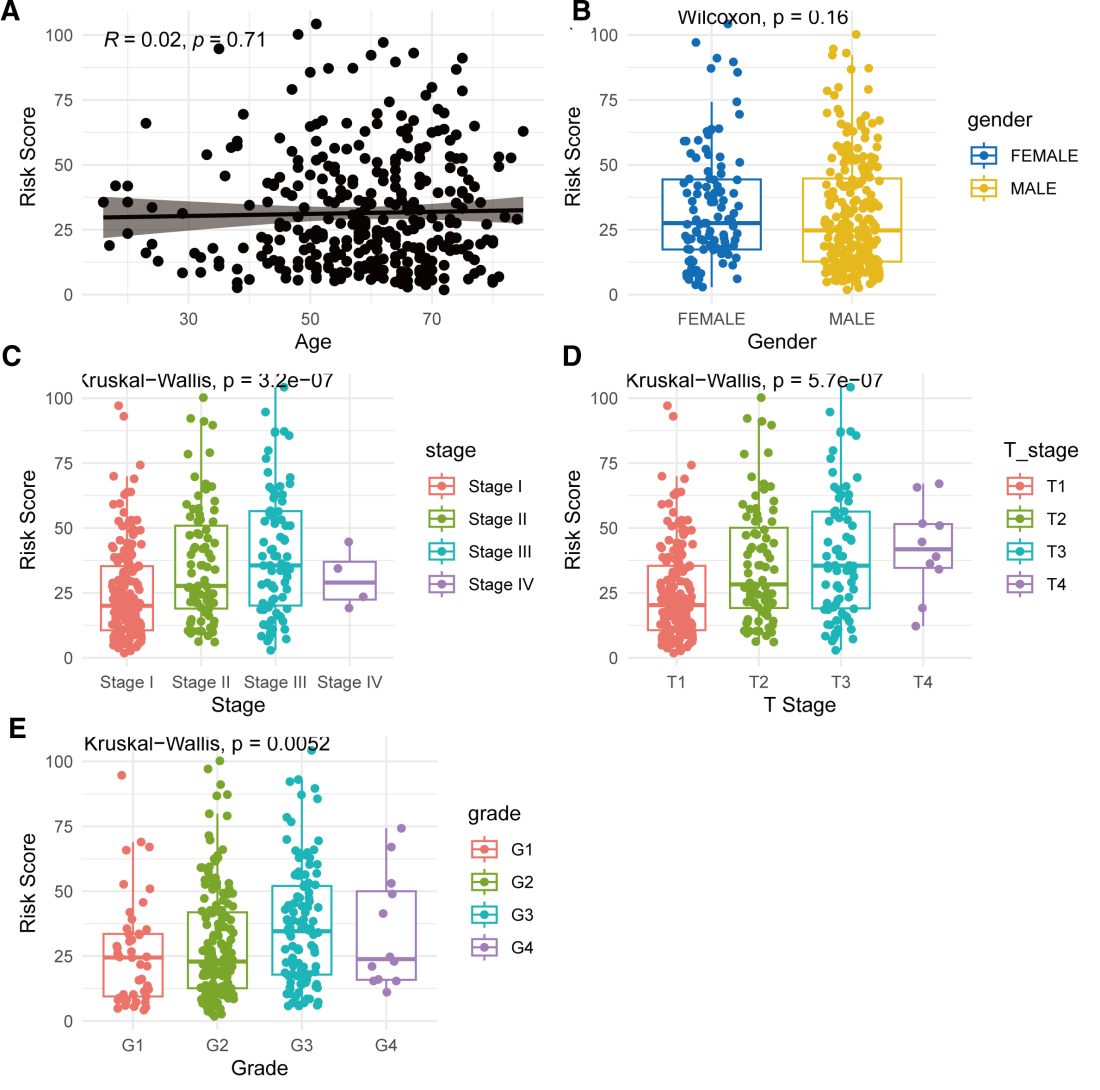
Correlation between risk score and clinicopathological features. (A) Scatter plot of risk score versus patient age (Spearman’s *R* = 0.02, *P* = .71). (B) Violin plot of risk score by gender. (C–E) Violin plots of risk score stratified by overall stage (C), T stage (D) and histologic grade (E). Statistical comparisons were performed using Kruskal–Wallis or Wilcoxon tests.

We next examined the correlation between RS and key clinical variables in TCGA-LIHC. Spearman correlation revealed no significant association between RS and patient age (*R* = 0.02, *P* = .71; Figure 3A). Likewise, RS did not differ by gender (Wilcoxon *P* = .16; Figure 3B). In contrast, RS was significantly higher in advanced overall stage (Kruskal–Wallis *P* = 3.2 × 10^−7^; Figure 3C), T stage (*P* = 5.7 × 10^−7^; Figure 3D) and higher histologic grade (*P* = .0052; Figure 3E), indicating that patients with more aggressive disease exhibited elevated RS.

### 3.4. Relationship between RS and tumor immune microenvironment

**Figure 4.**
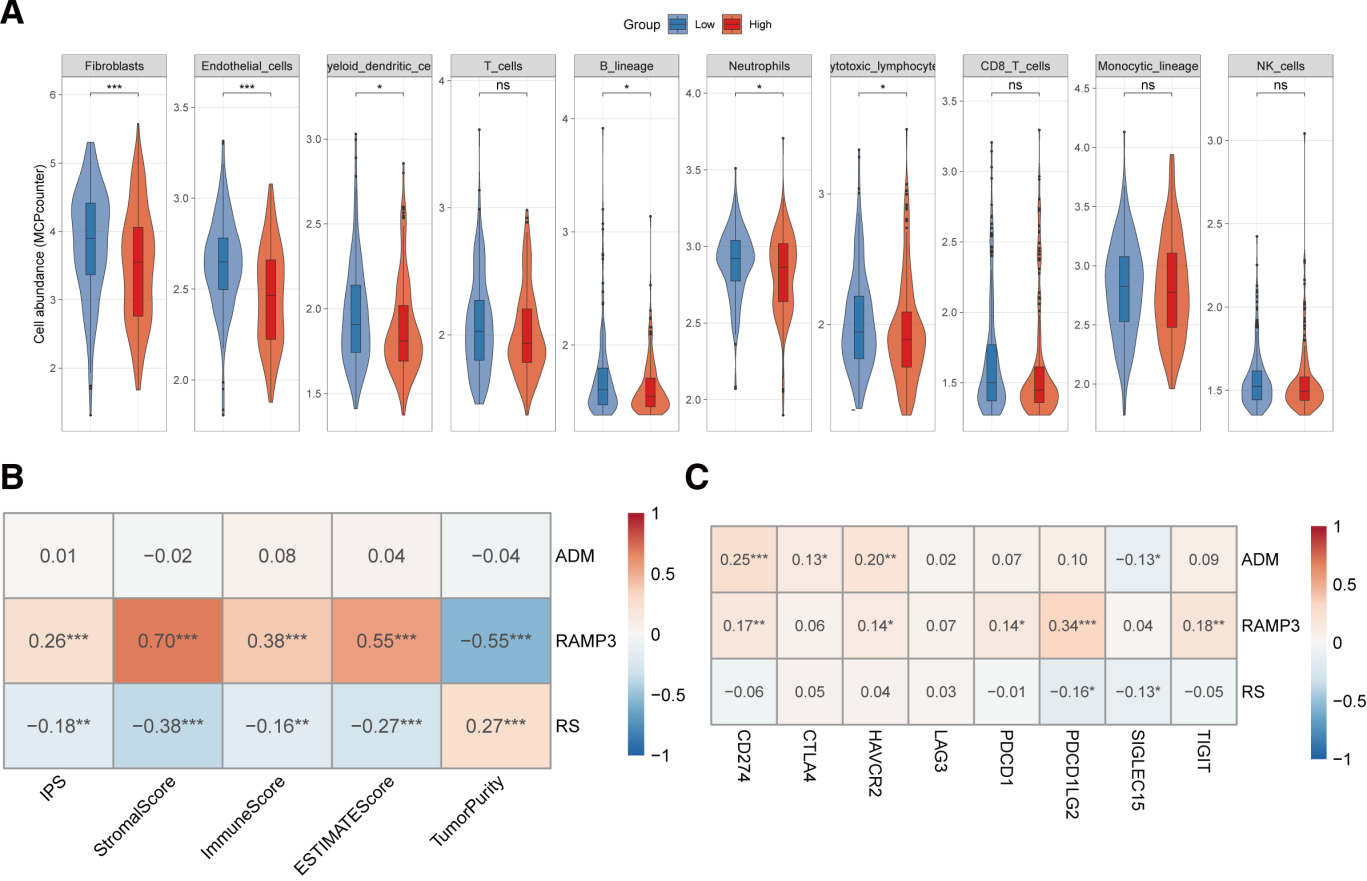
Association of risk score and ARG expression with the tumor immune microenvironment. (A) Differential infiltration levels of immune and stromal cell types between High- and Low-risk groups. (B) Heatmap of Spearman correlation coefficients between risk score, *RAMP3* expression and IPS, stromal, immune and ESTIMATE scores, as well as tumor purity. (C) Heatmap of correlations between ARGs/risk score and immune checkpoint gene expression. *P < .05; **P < .01; ***P < .001; ****P < .0001. IPS = Immunophenoscore, RS = risk score.

To explore the immunological correlates of our ARG-derived RS, we quantified immune cell infiltration, IPS and ESTIMATE scores in TCGA-LIHC. Compared with the Low group, the High group showed significantly reduced infiltration of endothelial cells (FDR = 1.4 × 10^−8^), fibroblasts (FDR = 5.8 × 10^−4^), myeloid dendritic cells (FDR = 0.022), neutrophils (FDR = 0.022), cytotoxic lymphocytes (FDR = 0.027), B cells (FDR = 0.027) and NK cells (FDR = 0.027) by MCP-counter (Fig. 4A). *RAMP3* expression correlated positively with stromal score (*R* = 0.70, FDR < 1 × 10^−12^), ESTIMATE score (*R* = 0.55, FDR < 1 × 10^−12^), immune score (*R* = 0.38, FDR = 1.3 × 10^−12^) and IPS (*R* = 0.26, FDR = 2.2 × 10^−6^), and negatively correlated with tumor purity (*r* = −0.55, FDR < 1 × 10^−12^); RS exhibited inverse associations (stromal score: *r* = −0.38, FDR = 1.8 × 10^−12^; ESTIMATE score: *r* = −0.27, FDR = 7.8 × 10^−7^; Figure 4B). Among immune checkpoint genes, *ADM* expression was positively associated with CD274 (*R* = 0.25, FDR = 3.4 × 10^−5^) and HAVCR2 (*R* = 0.20, FDR = 0.002); *RAMP3* correlated positively with PDCD1LG2 (*R* = 0.34, FDR = 3.4 × 10^−9^), TIGIT (*R* = 0.18, FDR = 0.004), PDCD1 (*R* = 0.14, FDR = 0.029) and CD274 (*R* = 0.17, FDR = 0.010); RS was inversely correlated with PDCD1LG2 (*r* = −0.16, FDR = 0.014) and SIGLEC15 (*r* = −0.13, FDR = 0.045; Figure 4C). To assess the robustness of immune infiltration estimates, a sensitivity analysis comparing MCP-counter with CIBERSORT (absolute mode) confirmed consistent directionality for CD8^+^ T cells across both algorithms (MCP-counter: median difference − 0.053, *P* = .043; CIBERSORT: −0.022, *P* = .042). Parallel immune deconvolution analysis in GSE14520 using MCP-counter confirmed consistent directionality of immune infiltration differences between high- and low-risk groups for CD8^+^ T cells and B cells (Supplementary Figure S2, Supplemental Digital Content 2). It should be noted that all immune infiltration estimates presented here are computationally inferred from bulk transcriptomic data using the MCP-counter algorithm and are therefore subject to the inherent limitations of deconvolution approaches, including inability to resolve spatial context, sensitivity to gene expression noise, and potential confounding by tumor cell–intrinsic transcriptomic signatures.

### 3.5. Correlation between RS and drug sensitivity

**Figure 5.**
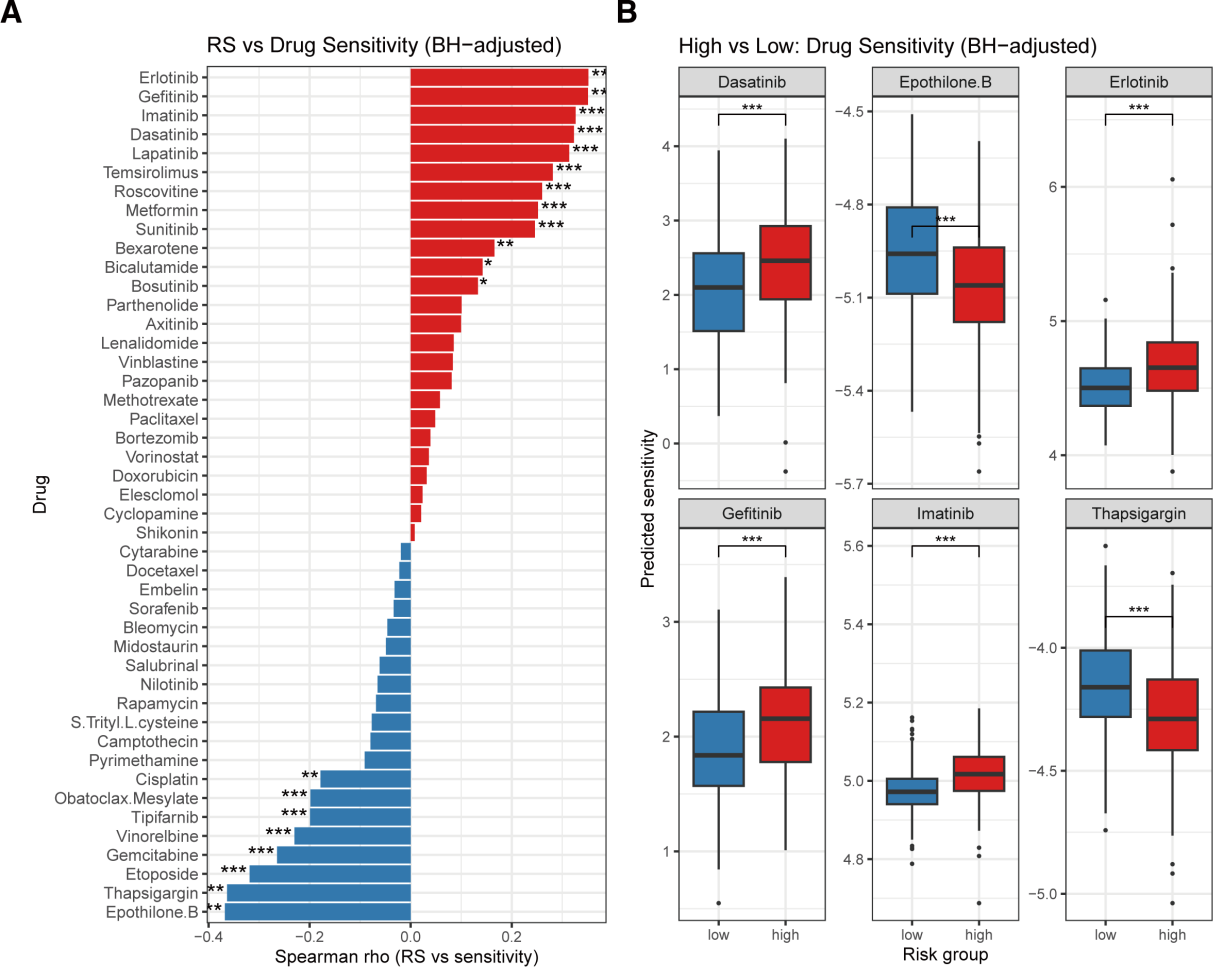
Computational prediction of drug sensitivity associations with risk score (in silico analysis; not validated by pharmacological assay). (A) Distribution of Spearman correlation coefficients between risk score and predicted sensitivity to 45 anticancer agents (FDR corrected). (B) Comparison of predicted sensitivities for the top 6 most correlated drugs in High- vs Low-risk groups. *P < .05; **P < .01; ***P < .001; ****P < .0001.

In silico drug sensitivity analysis using pRRophetic predicted that high-risk patients may exhibit greater sensitivity to certain targeted agents. RS was positively correlated with sensitivity to 12 agents including gefitinib, erlotinib and dasatinib, and negatively correlated with sensitivity to 8 agents including thapsigargin and etoposide (Fig. 5A). Six drugs with the strongest RS correlations were further compared between High- and Low-groups: dasatinib, erlotinib, gefitinib and imatinib sensitivities were significantly higher in High patients, whereas epothilone B and thapsigargin sensitivities were significantly lower (Fig. 5B). Cross-validation using the GDSC2 database via oncoPredict confirmed consistent directional predictions for erlotinib (pRRophetic: *R* = 0.30, FDR = 1.8 × 10^−7^; GDSC2: *R* = 0.12, FDR = 0.037) and gefitinib (pRRophetic: *R* = 0.27, FDR = 7.6 × 10^−7^; GDSC2: *R* = 0.15, FDR = 0.018), while dasatinib showed a consistent but nonsignificant trend in GDSC2 (*R* = 0.07, FDR = 0.17; Supplementary Figure S3, Supplemental Digital Content 3). These findings are associative and hypothesis-generating; they are not supported by pharmacological assays or patient-level treatment response data, and should be interpreted with caution.

### 3.6. Signaling pathways associated with RS

**Figure 6.**
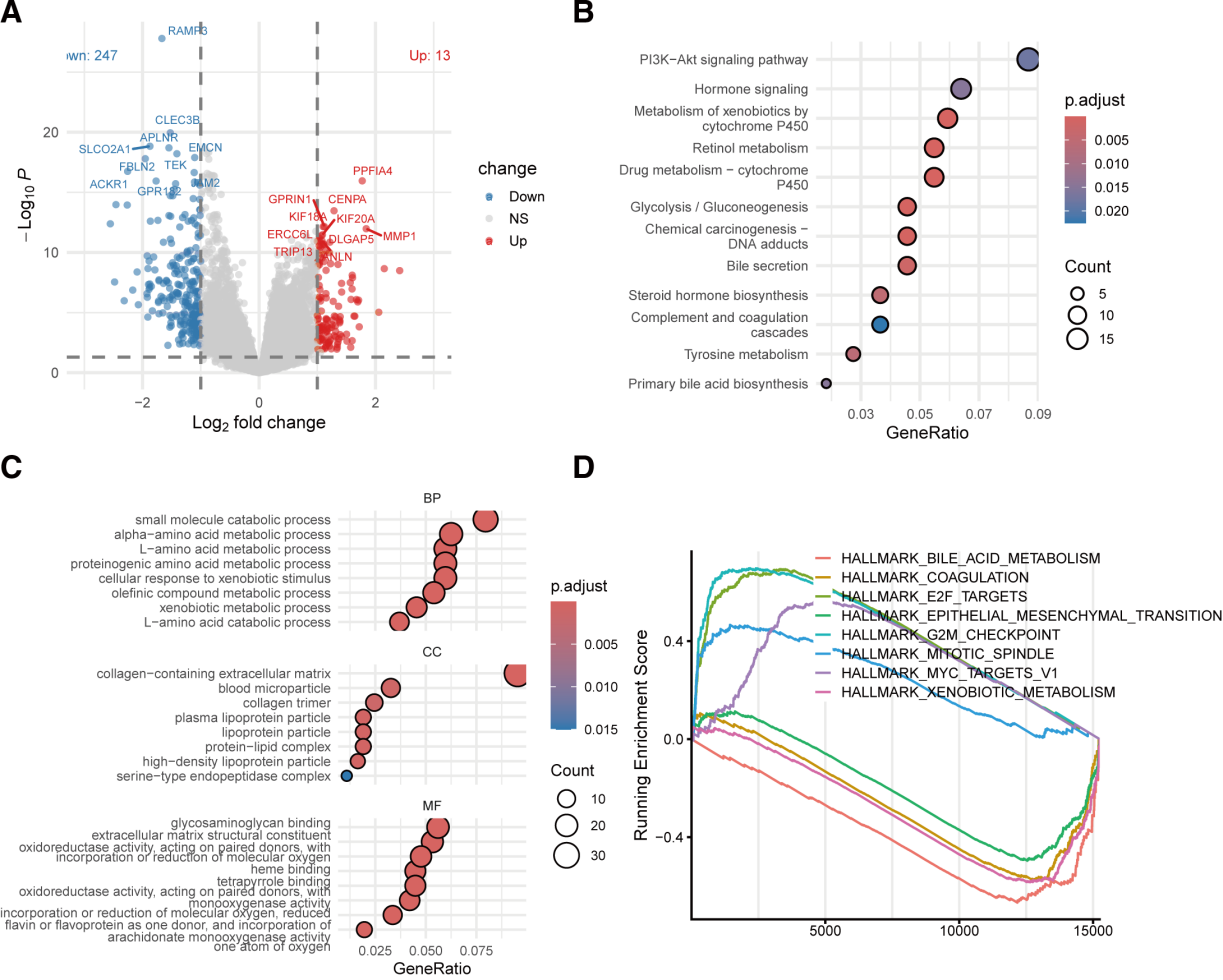
Pathway enrichment associated with risk score. (A) Volcano plot of differentially expressed genes between High- and Low-risk groups (|log_2_FC| > 1, adj. *P* < .05; 130 upregulated in red, 247 downregulated in blue). (B) GO enrichment dot plot (top 8 terms per ontology, FDR corrected). (C) KEGG pathway enrichment (FDR corrected). (D) GSEA of Hallmark gene sets showing top activated (G2M checkpoint, E2F targets, MYC targets V1, mitotic spindle) and suppressed (bile acid metabolism, xenobiotic metabolism, coagulation, EMT) pathways in the High-risk group.

Differential expression analysis between High and Low groups (|log_2_FC| > 1, adj. *P* < .05) identified 130 upregulated and 247 downregulated genes (Fig. 6A). The most significantly downregulated gene in the High-risk group was *RAMP3* (log_2_FC = −1.67, FDR = 2.4 × 10^−24^), consistent with its protective prognostic role. Gene Ontology enrichment analysis highlighted amino acid metabolic processes (FDR = 1.1 × 10^−8^), cellular response to xenobiotic stimulus (FDR = 1.5 × 10^−7^) and small molecule catabolic processes (FDR = 2.6 × 10^−7^; Figure 6B). KEGG pathway analysis identified enrichment in metabolism of xenobiotics by cytochrome P450 (FDR = 6.5 × 10^−6^), retinol metabolism (FDR = 6.5 × 10^−6^) and drug metabolism–cytochrome P450 (FDR = 8.4 × 10^−6^; Figure 6C). Gene set enrichment analysis of the Hallmark collection revealed that cell proliferation–related pathways: including G2M checkpoint (NES = 2.96, FDR = 0.001), E2F targets (NES = 2.95, FDR = 0.001), MYC targets V1 (NES = 2.38, FDR = 0.001) and mitotic spindle (NES = 1.98, FDR = 0.001): were significantly activated in the High-risk group (all FDR < 0.01). In contrast, metabolic and microenvironment-related pathways: including bile acid metabolism (NES = −2.41, FDR = 0.001), xenobiotic metabolism (NES = −2.26, FDR = 0.001), coagulation (NES = −2.12, FDR = 0.001) and epithelial-mesenchymal transition (NES = −1.93, FDR = 0.001): were significantly suppressed in the High-risk group (Fig. 6D). The transcriptomic profile of the High-risk group is thus characterized by enhanced proliferative and cell-cycle transcriptional programmes coupled with diminished metabolic and detoxification pathway activity.

### 3.7. Association between RS and somatic mutation burden

**Figure 7.**
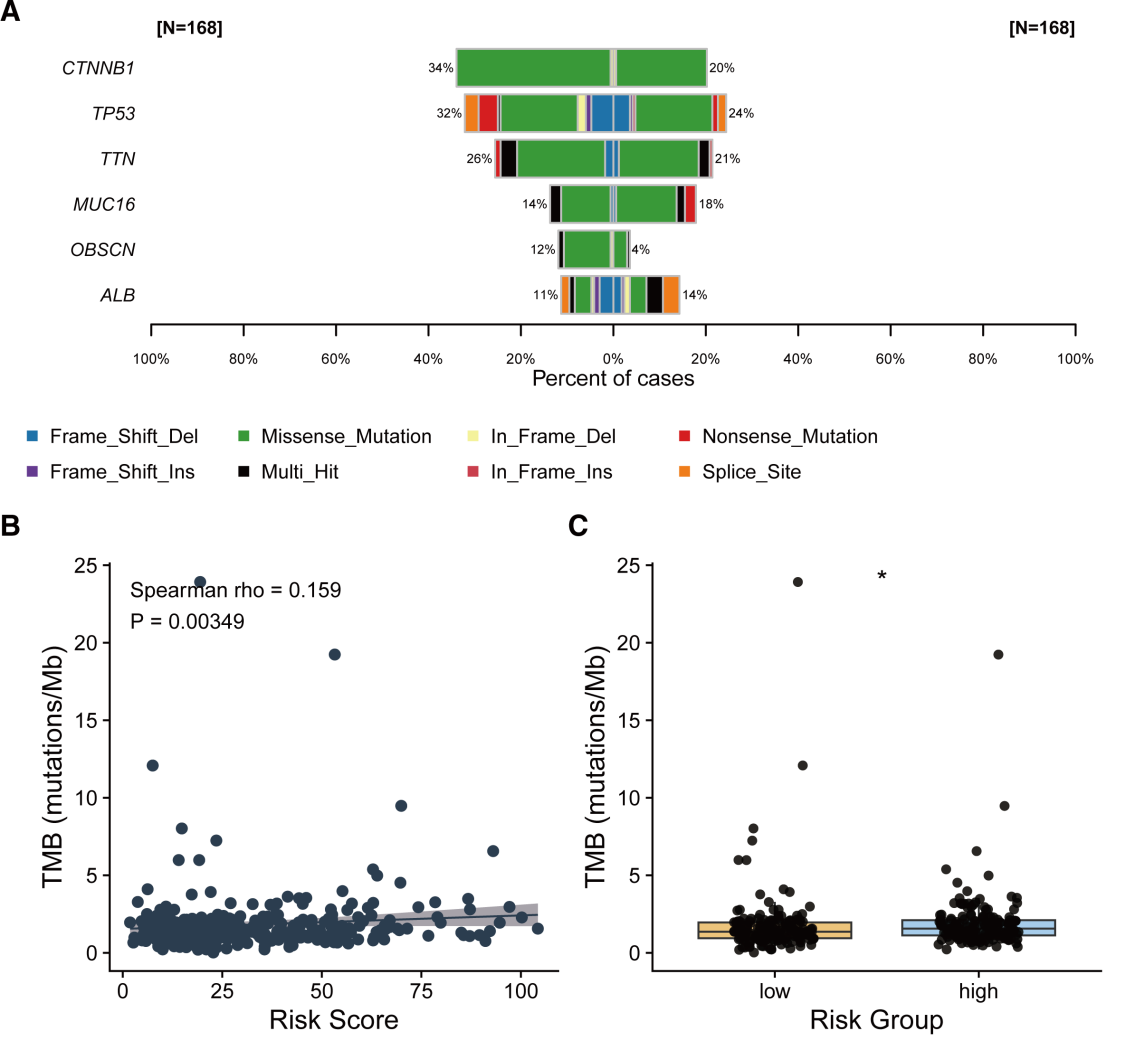
Association of risk score with somatic mutation frequency. (A) Oncoplot comparing mutation frequencies in High- vs Low-risk groups. (B) Scatter plot of risk score versus tumor mutational burden (TMB; Spearman’s *R* = 0.16, *P* = .0035). (C) Boxplot of TMB in High- and Low-risk groups (Wilcoxon *P* = .016). *P < .05. TMB = tumor mutational burden.

Somatic mutation profiles were compared between High and Low-risk groups. The most frequently mutated gene was TP53, detected in 32.1% of High-risk and 24.7% of Low-risk patients; however, this difference did not reach statistical significance after adjusting for T stage and histologic grade in logistic regression (adjusted OR = 1.26, 95% CI: 0.75–2.11, *P* = .38; Figure 7A). No individual gene showed a significant difference in mutation frequency between groups after FDR correction. RS was positively correlated with tumor mutational burden (TMB; Spearman’s *R* = 0.16, *P* = .0035; Figure 7B), and the High-risk group exhibited moderately elevated TMB relative to the Low-risk group (mean: 1.89 vs 1.75 mutations/Mb; Wilcoxon *P* = .016; Figure 7C).

### 3.8. Development of an ARG-based nomogram

**Figure 8.**
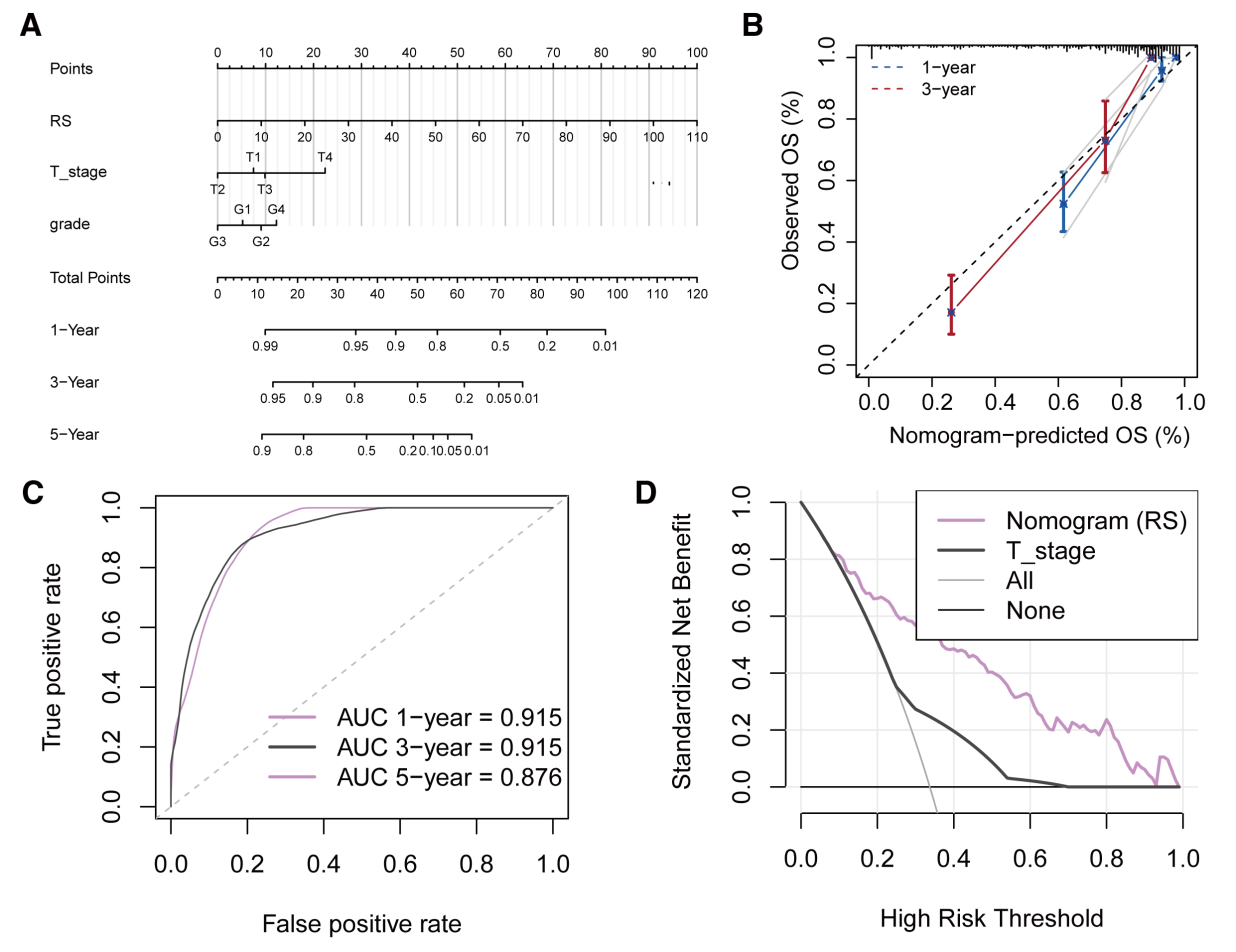
Nomogram for predicting OS in LIHC. (A) Nomogram incorporating risk score, T stage and histologic grade to estimate 1-, 3- and 5-year OS probabilities. (B) Calibration curves for 1- and 3-year OS. (C) Decision curve analysis comparing the combined model (RS + T stage + grade) with RS alone, T stage alone and grade alone. (D) Time-dependent ROC curves for 1-, 3- and 5-year OS (AUCs: 0.915/0.915/0.876; training cohort). LIHC = liver hepatocellular carcinoma, OS = overall survival, RS = risk score.

We next constructed a prognostic nomogram integrating the ARG-derived RS with clinical variables to facilitate individualized survival estimation. In univariate Cox analysis, RS (continuous, per unit increase) was the strongest predictor of OS (HR = 1.06, 95% CI:1.05–1.07, *P* = 4.7 × 10^−49^; Table 2). In multivariate Cox regression including RS, gender, age, T stage and histologic grade (overall stage excluded to avoid multicollinearity), RS remained the dominant independent predictor (HR per unit = 1.07, 95% CI: 1.06–1.08, *P* = 6.1 × 10^−41^), while T stage (HR for T4 vs T1 = 3.00, 95% CI: 1.31–6.86, *P* = .002) and histologic grade (overall *P* = .026) also retained independent significance (Table 2). A nomogram incorporating RS, T stage and histologic grade was therefore constructed for individualized prediction of 1-, 3-, and 5-year OS (Fig. 8A). Calibration plots demonstrated excellent agreement between nomogram-predicted and observed survival (Fig. 8B). DCA showed that the combined model (RS + T stage + grade) provided greater net clinical benefit than RS alone, T stage alone, or grade alone across a wide range of threshold probabilities (Fig. 8C). Time-dependent ROC analysis yielded AUCs of 0.915, 0.915 and 0.876 for 1-, 3- and 5-year OS, respectively (Fig. 8D). As these AUCs are computed on the training cohort, they represent resubstitution estimates; external validation is warranted.

**Table 2:** Univariate and multivariate cox regression analyses identified prognostic factors of LIHC.

Characteristics	Univariate cox		Multivariate cox	
	HR (95%CI)	*P*-value	HR (95%CI)	*P*-value
RS	1.06 (1.05–1.07)	4.7e-49	1.07 (1.06–1.08)	6.1e-41
Gender	1.30 (0.89–1.89)	.18	0.95 (0.63–1.42)	.8
Age	1.01 (1.00–1.02)	.17	1.01 (1.00–1.03)	.11
T_stage	5.07 (2.26–11.41)	3.5e-06	3.00 (1.31–6.86)	.0022
Grade	1.72 (0.62–4.81)	.76	1.63 (0.55–4.88)	.026

### 3.9. Single-cell heterogeneity of ARGs in HCC

**Figure 9.**
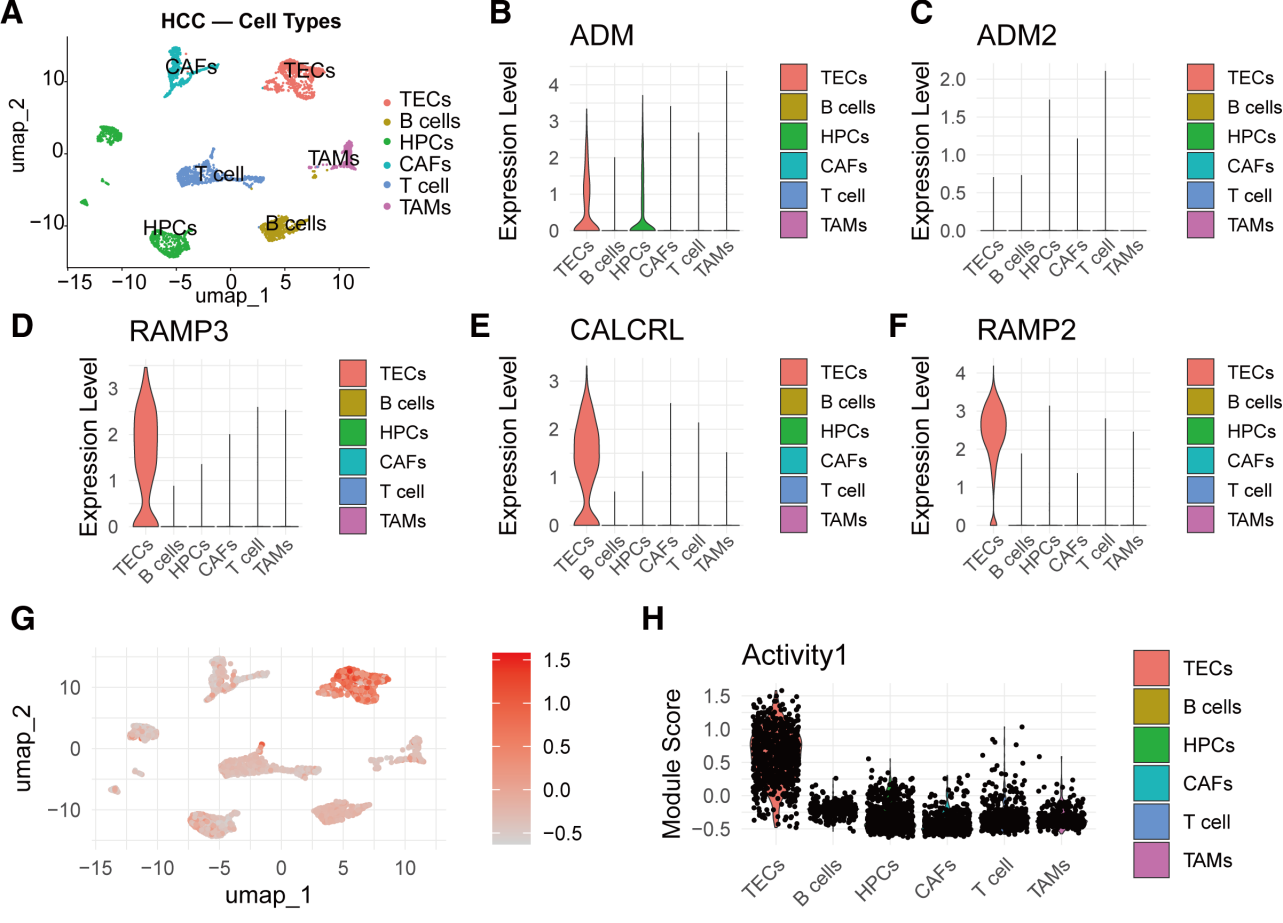
Single-cell heterogeneity of ARG expression in HCC. (A) UMAP plot showing 6 annotated cell populations after Harmony batch correction. (B–F) UMAP feature plots and violin plots of (B) *RAMP2*, (C) *RAMP3*, (D) *CALCRL*, (E) *ADM* and (F) *ADM*2 expression across cell types. (G–H) ARG pathway activity score (AddModuleScore) projected onto UMAP (G) and compared across cell types by violin plot (H). *** *P* < .001; Wilcoxon rank-sum test. ARGs = adrenomedullin receptor signaling-related genes, HCC = hepatocellular carcinoma.

To further characterize ARG expression heterogeneity at single-cell resolution, we reanalyzed the GSE125449 scRNA-seq dataset. Following quality control, doublet removal, Harmony batch correction and graph-based clustering, 6 major cell populations were annotated (Figure 9A, Supplementary Figure S4, Supplemental Digital Content 4): T cells, B cells, tumor-associated macrophages, cancer-associated fibroblasts (CAFs), tumor endothelial cells (TECs) and HPCs. Among the 5 ARGs,

*RAMP2*, *RAMP3* and *CALCRL* showed striking enrichment in TECs (mean expression: 2.43, 1.51 and 1.27, respectively; 93.2%, 75.4% and 74.3% of TECs positive; all *P* < 1 × 10^−100^ vs other cell types; Figure 9B-D, Supplementary Figure S5, Supplemental Digital Content 5). *ADM* was enriched in TECs (mean = 0.63, 43.5% positive) and, to a lesser extent, in HPCs (mean = 0.34; *P* = 1.5 × 10^−63^ vs other cells), whereas *ADM*2 was expressed at negligible levels across all cell types (Figs. 9E-F). Notably, *RAMP3*: one of the 2 core effectors in our prognostic model: exhibited near-exclusive endothelial expression (TEC mean = 1.51 vs 0.06 in all other cells combined), suggesting that the ARG-derived RS may partly reflect tumor vascular biology. ARG pathway activity scores (AddModuleScore) confirmed TECs as the dominant ARG-active population (mean score = 0.61; *P* = 6.9 × 10^−123^vs. CAFs, the next highest; Figure 9G-H).

## 4. Discussion

In this study, we developed and validated a novel prognostic signature for HCC based on ARGs. By integrating transcriptomic profiles and clinical data from the TCGA-LIHC and GSE14520 cohorts, and evaluating 116 combinations of machine learning algorithms, we identified a 2-gene model: *ADM* and *RAMP3*, constructed via RSF. Several multi-gene prognostic signatures for HCC have been established using distinct biological frameworks. Immune-related gene signatures, such as a T-cell exhaustion-based model^[24]^ and an immune-metabolism integrated signature,^[25]^ have demonstrated prognostic value by capturing the tumor–immune interplay. Ferroptosis-related gene signatures have also been developed,^[26]^ linking iron-dependent cell death pathways to HCC prognosis. Additionally, an autophagy-related gene signature^[27]^ and a medication decision gene signature^[19]^ have been proposed for risk stratification in HCC. Despite these advances, most existing signatures are derived from broad gene sets encompassing hundreds to thousands of genes across entire biological pathways, which may dilute mechanistic specificity and limit clinical translatability. In contrast, our model is built exclusively from 5 ARGs and is driven by only 2 core effectors (*ADM* and *RAMP3*), providing focused biological interpretability within a well-defined endocrine signaling axis. However, direct head-to-head comparison of predictive performance across models is limited by differences in cohort composition, follow-up duration, and outcome definitions; standardized benchmarking frameworks would be valuable for future comparative evaluations.

In contrast to prior prognostic signatures that incorporate broad pathway or tumor–microenvironment features, our model uniquely centers on the adrenomedullin receptor signaling axis by integrating its 5 core components. AM and its paralog *ADM*2 signal through the *CALCRL* in complex with receptor activity–modifying proteins *RAMP2* and *RAMP3* to regulate angiogenesis, proliferation and migration in multiple cancer types.^[10,28]^ For instance, in prostate cancer, AM stimulates the cAMP/CRAF/MEK/ERK cascade to drive tumor cell growth and promote tumor‐associated angiogenesis and lymphangiogenesis^[29]^; in glioblastoma, hypoxia‐induced AM expression correlates with enhanced neovascularization^[30]^; and in acute myeloid leukemia, *CALCRL* overexpression predicts poor outcome and chemoresistance.^[31,32]^ In the LIHC cohorts, *ADM* and *RAMP3* emerged as significant predictors of OS, implicating them in HCC progression and therapeutic resistance. Moreover, both univariate and multivariate Cox regression analyses confirmed the ARG‐derived RS as an independent prognostic factor, underscoring the potential of this pathway‐driven signature for refined risk stratification in LIHC. Notably, *RAMP3*: one of the 2 model genes in our RSF signature, showed near-exclusive expression in TECs (present in 75% of endothelial cells vs <3% in other populations), suggesting that the prognostic signal captured by the ARG RS may be partially driven by tumor vascular biology reflected in bulk transcriptomic data.

Immune microenvironment profiling using computational deconvolution suggested that the High-risk group exhibited reduced infiltration of multiple immune and stromal cell populations, including CAFs, endothelial cells, B cells and dendritic cells, relative to the Low-risk group. Concordantly, the ARG-derived RS was inversely correlated with ESTIMATE-derived stromal and immune scores and positively correlated with tumor purity, a pattern computationally consistent with an immunologically “cold” microenvironment. Moreover, expression of *ADM* and *RAMP3* correlated positively with key immune checkpoint genes such as PDCD1, CTLA4 and TIGIT, raising the possibility that AM signaling may be associated with checkpoint pathway modulation. These observations align with prior reports that AM promotes endothelial migration and tube formation^[28]^ and mediates immunosuppression and stem‐like phenotypes in acute myeloid leukemia.^[33]^ However, because these findings are derived from algorithmic inference rather than direct immunohistochemical or flow cytometric measurement of tumor-infiltrating lymphocytes, future studies employing multiplex immunohistochemistry, mass cytometry (CyTOF), or spatial transcriptomics would be necessary to directly validate the immune microenvironment composition in ARG risk-stratified HCC patients.

Somatic mutation analysis showed that TP53 mutation frequency was modestly higher in the High-risk group (32.1% vs 24.7%), though the difference was attenuated and no longer significant after adjustment for clinical covariates (OR = 1.26, *P* = .38). RS was weakly but significantly correlated with TMB (*R* = 0.16, *P* = .004), suggesting a modest association between ARG-based risk and genomic instability that is largely explained by clinical stage and grade. These findings indicate that the ARG-derived RS primarily captures proliferative and microenvironmental features rather than serving as a strong surrogate for mutational burden. In silico drug sensitivity predictions revealed that high‐risk patients were predicted to exhibit greater sensitivity to certain tyrosine kinase inhibitors (gefitinib, erlotinib, dasatinib) and less sensitivity to agents such as etoposide and thapsigargin. It is important to note that the drug sensitivity predictions derived from pRRophetic are based on pharmacogenomic data from cancer cell lines and should not be directly extrapolated to patient-level responses. This methodology carries inherent limitations including differences in tumor heterogeneity, microenvironmental context, and drug bioavailability between cell line models and patient tumors. Independent pharmacogenomic validation using resources such as GDSC2 or CTRP, as well as prospective clinical correlation studies, are necessary before these predictions can inform therapeutic decision-making. This drug‐response profile echoes mechanistic insights from other malignancies: for example, *ADM*2‐driven PKA/ERK activation in thyroid cancer^[34]^ and Src/c‐Myc–mediated translational upregulation in breast cancer^[35]^: and provides a rationale for further investigation of ARG-guided therapeutic strategies in LIHC, pending prospective validation.

Furthermore, our single-cell transcriptomic analysis demonstrated a pronounced enrichment of ARG expression and pathway activity in TECs within HCC lesions, implicating the AM signaling axis in the orchestration of tumor angiogenesis. These observations are consistent with prior studies showing that AM binds *CALCRL* in complex with *RAMP2* or *RAMP3* to drive endothelial cell migration and tube formation and synergizes with vascular endothelial growth factor to augment neovascularization.^[28]^ Endothelial-specific ablation of *RAMP2* has been reported to increase vascular permeability, elicit endothelial-to-mesenchymal transition–like changes, and promote metastatic dissemination, underscoring *RAMP2*’s critical role in maintaining vascular integrity and restraining tumor spread.^[36]^ Moreover, elevated expression of AM and its receptor components in HCC-associated endothelium has been correlated with advanced clinicopathological features.^[37]^ Collectively, these findings provide computational evidence suggesting a potential contribution of ARGs to HCC angiogenesis and immune microenvironment remodeling, warranting further investigation using spatially resolved and functionally validated experimental approaches before therapeutic strategies targeting the AM pathway can be rationally designed.

Despite these promising insights, our study has limitations. First, its retrospective design and reliance on public datasets warrant prospective, multicenter validation. Second, the mechanistic underpinnings of *ADM* and *RAMP3* in HCC biology remain to be elucidated through in vitro and in vivo experiments. Third, single-cell analyses were confined to a single dataset; larger cohorts are needed to confirm cell-type–specific ARG dynamics and their functional impacts. Fourth, cross-platform normalization between RNA-seq (TCGA) and microarray (GEO) data introduces potential inter-dataset variability that formal batch correction methods cannot fully address when datasets are used separately for training and validation. Future studies should validate the signature in prospectively collected cohorts using consistent RNA-seq platforms. Fifth, several secondary analyses, including drug sensitivity prediction, somatic mutation profiling, and detailed immune scoring, were conducted exclusively in TCGA-LIHC due to data availability constraints in GSE14520. While the prognostic performance of the ARG RS was validated in GSE14520, the biological correlates of the RS require replication in additional independent cohorts with complete multi-omic data. Sixth, the clinical variables available in TCGA-LIHC are limited, and important confounders such as etiology (hepatitis B/C vs metabolic dysfunction-associated steatohepatitis), treatment received, and liver function (Child–Pugh score) are not consistently available, potentially limiting the completeness of covariate adjustment. Future investigations should address these gaps, explore ARG modulation in preclinical HCC models, and assess the signature’s predictive value in the context of current systemic therapies.

## 5. Conclusion

We have presented the first adrenomedullin receptor signaling pathway–based prognostic model for HCC, comprising *ADM* and *RAMP3*, which robustly predicts patient survival and is associated with features consistent with an immunosuppressive microenvironment, differential predicted drug response, and increased mutation burden. This ARG-derived signature may serve as a hypothesis-generating framework for individualized prognosis prediction and for identifying potential therapeutic vulnerabilities in HCC that warrant prospective investigation. Further prospective validation and mechanistic studies are warranted to translate these findings into clinical application.

## Acknowledgments

The authors acknowledge the TCGA and GEO databases for providing publicly available data. No individuals are named in this section.

## Author contributions

**Conceptualization:** Dan Zhu.

**Methodology:** Dan Zhu.

**Software:** Dan Zhu.

**Data curation:** Siyi Zhong.

**Investigation:** Jiawei Hong.

**Visualization:** Jiawei Hong.

**Supervision:** Chicheng Lu.

**Writing – original draft:** Siyi Zhong.

**Writing – review & editing:** Li Zhuang.











## References

[1] Ahn HR Kim S Baek GO. Effect of Sortilin1 on promoting angiogenesis and systemic metastasis in hepatocellular carcinoma via the Notch signaling pathway and CD133. Cell Death Dis. 2024;15:634. 39209807 10.1038/s41419-024-07016-7 PMC11362463

[2] Liu D Li Q Zang Y. USP1 modulates hepatocellular carcinoma progression via the Hippo/TAZ axis. Cell Death Dis. 2023;14:264. 37041150 10.1038/s41419-023-05777-1 PMC10090121

[3] Oh H Kim J Jung SH. Discovery of 2,6-naphthyridine analogues as selective FGFR4 inhibitors for hepatocellular carcinoma. J Med Chem. 2024;67:8445–59. 38706130 10.1021/acs.jmedchem.4c00758

[4] Hwang SY Danpanichkul P Agopian V. Hepatocellular carcinoma: updates on epidemiology, surveillance, diagnosis and treatment. Clin Mol Hepatol. 2025;31(Suppl):S228–54. 39722614 10.3350/cmh.2024.0824 PMC11925437

[5] Nakamura T Masuda A Nakano D. Pathogenic mechanisms of metabolic dysfunction-associated steatotic liver disease (MASLD)-associated hepatocellular carcinoma. Cells. 2025;14:428. 40136677 10.3390/cells14060428 PMC11941585

[6] Guo X Zhao Z Zhu L. The evolving landscape of biomarkers for systemic therapy in advanced hepatocellular carcinoma. Biomark Res. 2025;13:60. 40221793 10.1186/s40364-025-00774-2 PMC11993949

[7] Lin HY Jeon A-J Chen K. The epigenetic basis of hepatocellular carcinoma - mechanisms and potential directions for biomarkers and therapeutics. Br J Cancer. 2025;132:869–87. 40057667 10.1038/s41416-025-02969-8 PMC12081707

[8] Qiu X Zhou T Li S. Spatial single-cell protein landscape reveals vimentin(high) macrophages as immune-suppressive in the microenvironment of hepatocellular carcinoma. Nat Cancer. 2024;5:1557–78. 39327501 10.1038/s43018-024-00824-y

[9] Ramirez CFA Akkari L. Myeloid cell path to malignancy: insights into liver cancer. Trends Cancer. 2025;11:591–610. 40140328 10.1016/j.trecan.2025.02.006

[10] Jailani ABA Bigos KJA Avgoustou P. Targeting the adrenomedullin-2 receptor for the discovery and development of novel anti-cancer agents. Expert Opin Drug Discov. 2022;17:839–48. 35733389 10.1080/17460441.2022.2090541

[11] Nakayama A Albarrán-Juárez J Liang G. Disturbed flow-induced Gs-mediated signaling protects against endothelial inflammation and atherosclerosis. JCI Insight. 2020;5:e140485. 33268595 10.1172/jci.insight.140485 PMC7714404

[12] Li H Yang W Wang S. Adrenomedullin in tumorigenesis and cancer progression. Int J Mol Sci. 2025;26:5552. 40565016 10.3390/ijms26125552 PMC12193685

[13] Bálint L Nelson-Maney NP Tian Y Serafin SD Caron KM. Clinical potential of adrenomedullin signaling in the cardiovascular system. Circ Res. 2023;132:1185–202. 37104556 10.1161/CIRCRESAHA.123.321673 PMC10155262

[14] Li YM Xu C Sun B Zhong F-J Cao M Yang L-Y. Piezo1 promoted hepatocellular carcinoma progression and EMT through activating TGF-β signaling by recruiting Rab5c. Cancer Cell Int. 2022;22:162. 35461277 10.1186/s12935-022-02574-2 PMC9035260

[15] Ma S Meng G Liu T. The Wnt signaling pathway in hepatocellular carcinoma: regulatory mechanisms and therapeutic prospects. Biomed Pharmacother. 2024;180:117508. 39362068 10.1016/j.biopha.2024.117508

[16] Ning J Ye Y Bu D. Imbalance of TGF-β1/BMP-7 pathways induced by M2-polarized macrophages promotes hepatocellular carcinoma aggressiveness. Mol Ther. 2021;29:2067–87. 33601054 10.1016/j.ymthe.2021.02.016 PMC8178441

[17] Shen H Li H Tang H. CCDC110 promotes the progression of hepatocellular carcinoma by activating the TGF-β/SMAD signaling pathway through targeted regulation of TGFBR1. Cancer Cell Int. 2025;25:183. 40394630 10.1186/s12935-025-03803-0 PMC12093845

[18] Rezende Miranda R Fu Y Chen X. Development of a potent and specific FGFR4 inhibitor for the treatment of hepatocellular carcinoma. J Med Chem. 2020;63:11484–97. 33030342 10.1021/acs.jmedchem.0c00044

[19] Yuan J Liu Z Wu Z Yan L Yang J Shi Y. A novel medication decision gene signature predicts response to individualized therapy and prognosis outcomes in hepatocellular carcinoma patients. Front Immunol. 2022;13:990571. 36275751 10.3389/fimmu.2022.990571 PMC9585274

[20] Ma L Hernandez MO Zhao Y. Tumor cell biodiversity drives microenvironmental reprogramming in liver cancer. Cancer Cell. 2019;36:418–30.e6. 31588021 10.1016/j.ccell.2019.08.007 PMC6801104

[21] McGinnis CS Murrow LM Gartner ZJ. DoubletFinder: doublet detection in single-cell RNA sequencing data using artificial nearest neighbors. Cell Syst. 2019;8:329–37.e4. 30954475 10.1016/j.cels.2019.03.003 PMC6853612

[22] Korsunsky I Millard N Fan J. Fast, sensitive and accurate integration of single-cell data with Harmony. Nat Methods. 2019;16:1289–96. 31740819 10.1038/s41592-019-0619-0 PMC6884693

[23] Aran D Looney AP Liu L. Reference-based analysis of lung single-cell sequencing reveals a transitional profibrotic macrophage. Nat Immunol. 2019;20:163–72. 30643263 10.1038/s41590-018-0276-y PMC6340744

[24] Chi H Zhao S Yang J. T-cell exhaustion signatures characterize the immune landscape and predict HCC prognosis via integrating single-cell RNA-seq and bulk RNA-sequencing. Front Immunol. 2023;14:1137025. 37006257 10.3389/fimmu.2023.1137025 PMC10050519

[25] Gu Y Ma E Jiang S. Immune- and metabolism-related gene signature analysis uncovers the prognostic and immune microenvironments of hepatocellular carcinoma. J Cancer Res Clin Oncol. 2024;150:311. 38896142 10.1007/s00432-024-05849-5 PMC11186947

[26] He Y Wu Y Song M Yang Y Yu Y Xu S. Establishment and validation of a ferroptosis-related prognostic signature for hepatocellular carcinoma. Front Oncol. 2023;13:1149370. 37143953 10.3389/fonc.2023.1149370 PMC10151679

[27] Ma Z Chen M Liu XL Cui H. Identification and verification of a prognostic autophagy-related gene signature in hepatocellular carcinoma. Sci Rep. 2024;14:3032. 38321105 10.1038/s41598-024-53565-4 PMC10847443

[28] Fernandez-Sauze S Delfino C Mabrouk K. Effects of adrenomedullin on endothelial cells in the multistep process of angiogenesis: involvement of CRLR/*RAMP2* and CRLR/*RAMP3* receptors. Int J Cancer. 2004;108:797–804. 14712479 10.1002/ijc.11663

[29] Berenguer-Daizé C Boudouresque F Bastide C. Adrenomedullin blockade suppresses growth of human hormone-independent prostate tumor xenograft in mice. Clin Cancer Res. 2013;19:6138–50. 24100627 10.1158/1078-0432.CCR-13-0691

[30] Metellus P Voutsinos-Porche B Nanni-Metellus I. Adrenomedullin expression and regulation in human glioblastoma, cultured human glioblastoma cell lines and pilocytic astrocytoma. Eur J Cancer. 2011;47:1727–35. 21458987 10.1016/j.ejca.2011.02.021

[31] Angenendt L Bormann E Pabst C. The neuropeptide receptor calcitonin receptor-like (*CALCRL*) is a potential therapeutic target in acute myeloid leukemia. Leukemia. 2019;33:2830–41. 31182782 10.1038/s41375-019-0505-x

[32] Larrue C Guiraud N Mouchel P-L. Adrenomedullin-*CALCRL* axis controls relapse-initiating drug tolerant acute myeloid leukemia cells. Nat Commun. 2021;12:422. 33462236 10.1038/s41467-020-20717-9 PMC7813857

[33] Gluexam T Grandits AM Schlerka A. CGRP Signaling via *CALCRL* increases chemotherapy resistance and stem cell properties in acute myeloid leukemia. Int J Mol Sci. 2019;20:5826. 31756985 10.3390/ijms20235826 PMC6928760

[34] Kim JT Lim MA Lee SE. Adrenomedullin2 stimulates progression of thyroid cancer in mice and humans under nutrient excess conditions. J Pathol. 2022;258:264–77. 36098211 10.1002/path.5997 PMC9826144

[35] Kong L Xiong Y Wang D. Intermedin (adrenomedullin 2) promotes breast cancer metastasis via Src/c-Myc-mediated ribosome production and protein translation. Breast Cancer Res Treat. 2022;195:91–103. 35896852 10.1007/s10549-022-06687-0

[36] Tanaka M Koyama T Sakurai T. The endothelial adrenomedullin-*RAMP2* system regulates vascular integrity and suppresses tumour metastasis. Cardiovasc Res. 2016;111:398–409. 27307317 10.1093/cvr/cvw166

[37] Cabiati M Gaggini M De Simone P. Expression profile of adrenomedullin and its specific receptors in liver tissues from patients with hepatocellular carcinoma and in tumorigenic cell line-secreted extracellular vesicles. Pathol Res Pract. 2023;243:154383. 36827885 10.1016/j.prp.2023.154383

